# Touch-Based Partner Yoga for Gay, Bisexual, Transgender, and Queer Men in a Community Wellness Setting: Protocol for a Mixed Methods Program Evaluation of “The Studio”

**DOI:** 10.2196/86310

**Published:** 2026-04-29

**Authors:** Jesse Strunk Elkins, Travis R Scheadler, Pasquale E Rummo

**Affiliations:** 1 Department of Epidemiology and Community Health, University of North Carolina at Charlotte, 9201 University City Blvd, Charlotte, NC, 28223-0001, United States, 1 704-687-8622; 2 School of Social Work, University of North Carolina at Charlotte, Charlotte, NC, United States; 3 Department of Population Health, NYU Grossman School of Medicine, New York City, NY, United States

**Keywords:** yoga, touch, physical activity, leisure, mixed methods research, sexual and gender minorities

## Abstract

**Background:**

Leisure-time physical activity (LTPA) is a well-established contributor to physical, psychological, and social well-being worldwide. Human touch also plays a vital role in life course health, yet opportunities for safe, consensual touch are often limited, particularly in LTPA settings. For gay, bisexual, transgender, and queer (GBTQ) men, barriers to affirming LTPA spaces can make it particularly difficult to access such benefits. In response, community-based approaches that integrate touch are needed, alongside systematic evaluations of such strategies. “The Studio” (pseudonym), a membership-based wellness community, addresses this gap by offering touch-centered partner yoga and bodywork programs designed to support the holistic health of GBTQ men.

**Objective:**

This protocol describes a mixed methods evaluation of the Studio’s touch-based yoga programming in New York City. The primary aim is to assess the feasibility and acceptability of implementing a touch-centered partner yoga program within a GBTQ community wellness setting. Secondary aims include exploring preliminary physical, emotional, and social outcomes associated with participation, including flexibility, stress, body awareness, social connection, trust, and belonging.

**Methods:**

The evaluation uses a pre- and posttest mixed methods design. A total of 40‐50 participants will be recruited from new Studio members. Quantitative measures will include flexibility (sit-and-reach and goniometry), stress (Perceived Stress Scale), body awareness (Multidimensional Assessment of Interoceptive Awareness), and resilience (Brief Resilience Scale). Social network analysis will map participant connections before and after program participation. Qualitative data will be collected through semistructured interviews with 15‐20 participants, or until saturation is reached, focusing on comfort with touch, emotional regulation, and experiences of community connectedness. Survey and interview guides will be codeveloped with a community advisory group to ensure cultural responsiveness and relevance. Findings will be integrated using triangulation methods to explore convergence across data sources.

**Results:**

As of March 2026, this study has not yet begun. Institutional Review Board submission is planned for September 2026. Afterward, study instruments will be finalized and pilot-tested with Studio teachers. Participant recruitment is projected to begin in July 2027, and data collection will include 3 time points (baseline, postintervention, and 4‐6 wk follow-up). Data analysis and dissemination of findings are expected in 2028. Preliminary pilot testing of the survey instruments with Studio employees and community advisory group members will indicate feasibility and cultural fit.

**Conclusions:**

This evaluation will be among the first to systematically examine touch-focused partner yoga for GBTQ men in a community wellness setting. Findings are expected to provide novel insights into the feasibility and the role of intentional touch in LTPA spaces, support trauma-informed and inclusive wellness practices, and contribute to broader discourse on GBTQ health promotion and intervention. Results will be disseminated to the Studio employees, members, and GBTQ-focused wellness organizations, as well as through peer-reviewed publications and conferences.

## Introduction

### Background

Leisure-time physical activity (LTPA) improves physical and mental health, lowering chronic disease and mortality risk while enhancing mood and well-being [1,2]. Consensual touch fosters belonging, reduces loneliness, and buffers stress, yet is often absent or stigmatized in structured physical activity spaces [3,4]. Touch reduces stress through neurobiological pathways, while LTPA independently strengthens physical and psychological health. Together, these exposures may produce synergistic health benefits [5,6]. However, the absence of touch is particularly harmful for adults who face systemic inequities in access to affirming touch and inclusive wellness spaces, shaped by race, sexuality, gender, age, income, and geography [7,8]. Marginalized groups, especially gay, bisexual, transgender, and queer (GBTQ) men, report lower LTPA participation and higher rates of depression, anxiety, and loneliness. Yet, no intervention has measured combined LTPA and touch strategies that may address these disparities [9-12].

Historically, research on LTPA and human touch has been developed along parallel tracks, despite growing evidence that each exposure independently influences psychosocial and physiological health. Touch-centered movement modalities, including partner yoga and massage-informed practices, integrate structured physical activity with consensual interpersonal contact and may activate complementary neurobiological pathways related to stress regulation, affective processing, and social bonding [5,13,14]. Consensual touch has been associated with reductions in loneliness, anxiety, and physiological stress responses, while LTPA is consistently linked to improved mood, reduced chronic disease risk, and enhanced well-being across diverse populations [2,15,16]. Emerging literature suggests that combining movement with intentional touch may strengthen intrinsic motivation and belonging through embodied and relational mechanisms [17,18]. However, empirical evaluation of these dual-exposure approaches remains limited, particularly within community-based settings for GBTQ individuals, highlighting a critical gap addressed by this study.

Importantly, within touch-centered LTPA, consent serves not only as an ethical safeguard but also as a foundational mechanism that shapes agency, safety, and empowerment. In consent-based somatic and trauma-informed frameworks, the negotiation of boundaries, participant choice, and relational attunement is an ongoing process, in which touch can be both therapeutic and a source of vulnerability, particularly for GBTQ individuals navigating stigma or exclusion within movement spaces [19,20]. Enthusiastic, clearly communicated consent transforms touch from passive contact into an active, embodied process that reinforces autonomy and psychological safety while supporting social connection and intrinsic motivation [14,18]. Positioning intentional touch through a consent-centered lens strengthens the theoretical coherence among the physiological benefits of touch, the psychosocial goals of LTPA, and the ethical and methodological safeguards described later in this protocol.

This study offers a research protocol designed to systematically examine touch-focused partner yoga for GBTQ men in a community wellness setting and posits this unique opportunity as an innovative, structured model integrating touch and LTPA. The project aims to test the feasibility and the physical and psychosocial benefits of touch-based LTPA, offering a replicable public health strategy scalable across diverse settings.

### Program Overview

“The Studio” (pseudonym) is a wellness community and online social platform dedicated to helping GBTQ men access touch-based LTPA for their physical, emotional, social, and spiritual well-being. Offering both in-person and online experiences, the Studio provides a range of practices, including bodywork education (eg, massage), yoga, energy healing, and spiritual rituals, as well as various immersive events. According to organizational materials, the Studio describes its programming as strongly focused on nurturing human connection, with intentional touch, structured classes, and social engagement coming together to support personal growth and collective healing.

The organization primarily serves GBTQ men and attracts individuals who are interested in wellness, bodywork, and the therapeutic benefits of touch, as well as those seeking structured and social opportunities for mindful movement, partner-based physical practices, and immersive well-being experiences. The Studio’s program materials describe multifaceted intended outcomes across physical, emotional, psychological, and social domains. Physically, participants may improve mobility and flexibility and achieve long-term muscle relaxation through partner-based yoga practices. Emotionally and psychologically, the Studio aims to enhance relaxation, alleviate stress, and increase emotional connection through safe consensual touch. Socially, the Studio aims to strengthen interpersonal bonds and promote a sense of belonging through shared movement, healing practices, and immersive events. Additionally, the Studio seeks to normalize healing touch within LTPA while contributing to a broader discourse on GBTQ men’s wellness. Limited, publicly available evaluation and implementation history relevant to this protocol is outlined in the following section to situate the program within its broader development trajectory.

### Program Development, Implementation, and Evaluation History

Since its creation, the Studio has engaged more than 10,000 members. At its New York City location, the Studio hosts private, member-only events that emphasize partner-based and touch-focused yoga classes. These classes integrate partner-based relaxation methods, muscle relief, and embodied connection, centering the power of intentional physical contact within a safer, more welcoming space. Although the Studio has a well-developed infrastructure and diverse programming, there is no indication of a formal evaluation history.

### Implementation, Population, Setting, and Geography

This protocol describes an evaluation of the Studio’s touch-based yoga classes within its membership-based wellness space in New York City. The city is a diverse, urban LGBTQ+ (lesbian, gay, bisexual, transgender, questioning, or queer and others) hub; the geographic focus offers a valuable opportunity to assess the Studio’s impact on broader populations in addition to GBTQ men [21].

### Evaluation Framework

**Figure 1.**
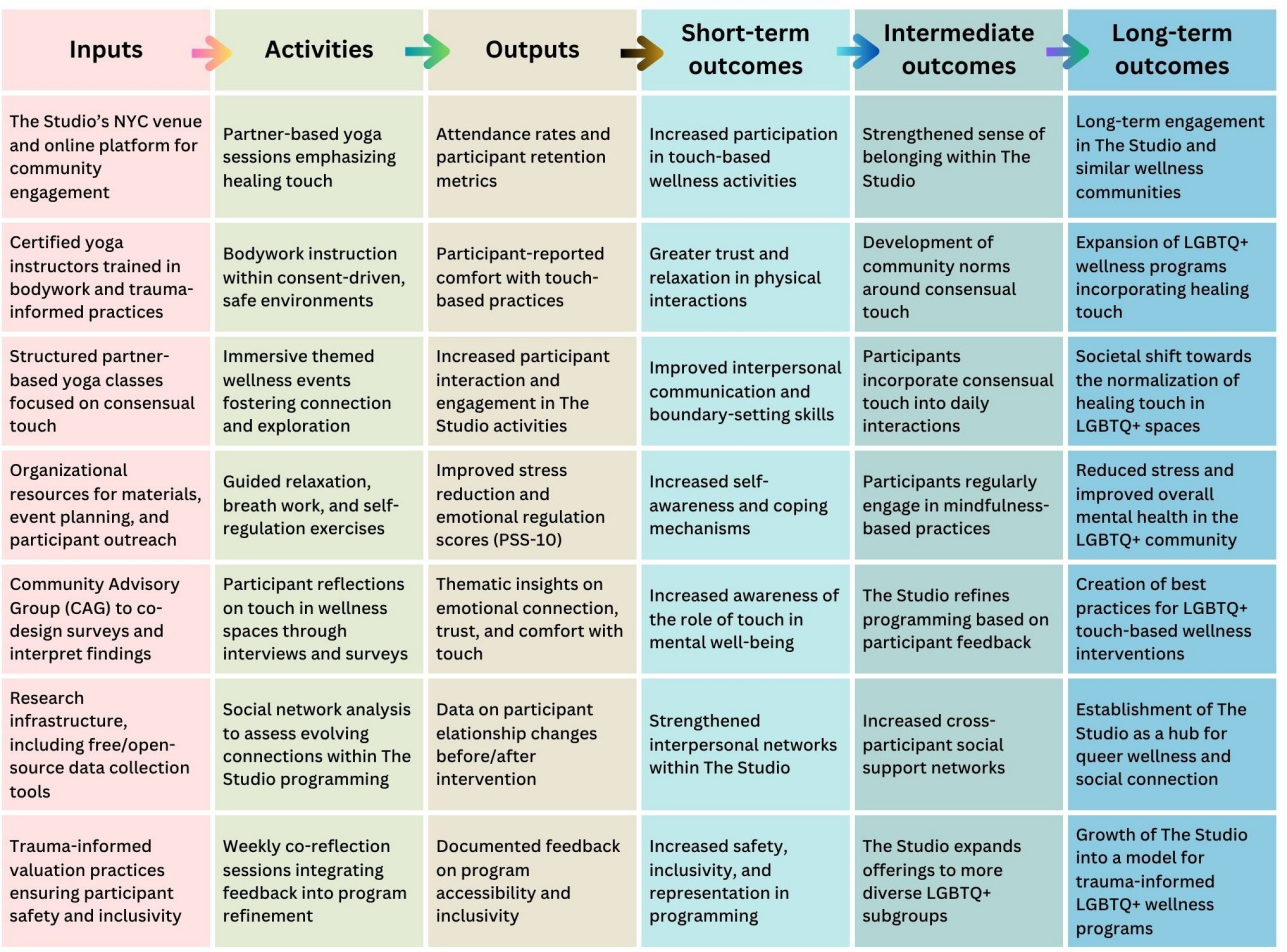
Logic model: studio program inputs, activities, outputs, and outcomes over time.

This evaluation uses a logic model framework (Figure 1) to systematically map out the inputs, activities, outputs, and intended outcomes of the Studio’s partner-based yoga programming [22]. Using a logic model for this evaluation is key to providing a structured visualization of multidimensional program components and to clearly connecting inputs to outcomes. Participatory logic modeling also enhances validity by incorporating community-based feedback into program design [23]. Figure 1 illustrates that the proposed evaluation is grounded in inputs such as the Studio’s New York City location and online community platform, certified yoga instructors trained in bodywork and trauma-informed practices, and structured partner-based yoga classes emphasizing consensual touch. These inputs are supported by organizational resources for materials and outreach, a community advisory group (CAG) to co-design instruments and interpret findings, research infrastructure (including free and open-source data collection tools), and trauma-informed evaluation practices to safeguard participant safety and inclusivity.

These resources enable a range of activities designed to foster connection and well-being. Inputs are operationalized through partner-based yoga sessions focused on healing touch, bodywork instruction within consent-driven environments, immersive wellness events, and guided practices, such as breathwork and self-regulation exercises. In addition, participant reflections will be gathered through interviews and surveys; social network analysis (SNA) will be used to assess evolving connections; and weekly coreflection sessions will allow for observational notes to contribute to program refinement. Together, these activities ensure that the Studio’s evaluation will be grounded in both community feedback and evidence-based practice.

The activities, in turn, generate measurable outputs that capture program reach and impact. These include attendance and retention metrics, results from pre-post flexibility and stress assessments, and qualitative insights from semistructured interviews highlighting themes, such as emotional connection and trust-building. Additional outputs include community engagement indicators from survey responses and network mapping data, as well as observed changes in comfort levels with intentional touch practices. By systematically linking outputs to the activities that produced them, the evaluation framework enables assessment of both immediate program effectiveness and broader community impact.

The logic model then connects outputs to a progression of outcomes over time. In the short term, participants are expected to demonstrate increased awareness of the benefits of touch in physical activity, improvements in flexibility and mobility, enhanced emotional regulation, and strengthened interpersonal trust. Intermediate outcomes extend this impact, including the normalization of healing touch in GBTQ wellness spaces, greater social cohesion, expanded programming that better reflects diverse GBTQ identities, and increased participant confidence in engaging in touch-based practices. Long-term outcomes focus on maintaining these gains by promoting broader acceptance of healing touch in GBTQ wellness and movement settings, enhancing overall quality of life, and encouraging ongoing engagement in intentional touch-based practices. Over time, the Studio may also adapt programming to be more trauma-informed and inclusive of participants beyond GBTQ men, thereby extending its impact and scalability.

In addition to the single flow depicted here, the model can also be viewed through 2 parallel trajectories—participant outcomes (eg, stress reduction, belonging, and long-term touch-positive LTPA) and program outcomes (eg, facilitator development, refinement of offerings, and long-term expansion). The outputs and outcomes depicted in the logic model are operationalized through specific quantitative and qualitative measures described in-depth later in this protocol. For example, short-term physical outcomes, such as flexibility and mobility, correspond to sit-and-reach and goniometry assessments; emotional regulation and stress-related outcomes are assessed using the Perceived Stress Scale-10 items (PSS-10), the Multidimensional Assessment of Interoceptive Awareness (MAIA), and the Brief Resilience Scale (BRS); and social and relational outcomes, such as belonging, trust, and community cohesion, are evaluated through SNA metrics (eg, network density and centrality) alongside qualitative interview themes. Mapping conceptual outcomes to measurable indicators ensures that the logic model directly informs data collection, analysis, and interpretation of the study’s key research questions.

### Community Engagement

Community engagement will involve multiple groups, including the Studio participants, employees, and the leadership team, ensuring that the evaluation is collaborative, community-informed, and aligned with the needs of members directly impacted. The primary participants include GBTQ men newly attending Studio classes, as well as the Studio teachers and organizational leaders who design and implement programming. Community engagement will be central to shaping the evaluation process, interpreting findings, and informing programmatic improvements.

To facilitate meaningful participation, a CAG will be established, consisting of 5‐10 Studio members (nonstudy participants) who will co-design and approve evaluation administration by participating in email communications, providing input on data collection instruments, and contributing to the interpretation of results following analysis [24,25]. The CAG will meet biweekly for 60 minutes each, and notes from each CAG session will be documented and reviewed by the research team to ensure community feedback is systematically incorporated into study processes. The CAG will remain active across the full evaluation lifecycle, beginning during instrument co-development before recruitment (anticipated May 2027) and continuing through data collection, preliminary interpretation, and dissemination planning (through approximately October 2028). Biweekly meetings are anticipated during the project’s active phases, with reduced frequency during analysis periods as needed. Based on the projected timeline, approximately 20‐30 meetings are expected during the evaluation. Because community participation may evolve over time, membership changes will be managed through a rolling participation model, with new members onboarded using the same orientation procedures to maintain continuity while preserving community representation. Equity and inclusivity will be prioritized by compensating CAG members with US $50 gift cards for each meeting attended (up to US $100 per month).

In addition to the CAG, Studio facilitators and leadership will participate in biweekly 60-minute coreflection sessions with the research team. These sessions will provide an opportunity to review study progress along with CAG reports and embed real-time reflection into the evaluation process. At the conclusion of the evaluation, Studio leaders may use such ongoing reflections to triangulate evaluation data to systematically integrate relevant improvements in member interactions, class offerings, and teaching styles. This multilevel engagement approach ensures that community members are active contributors, aligning with community-driven wellness.

### Evaluation Objectives and Questions

This evaluation has 2 primary objectives. The first objective is to assess feasibility and acceptability indicators, including recruitment rate (proportion of eligible participants enrolled), retention rate (proportion completing all study time points), attendance or adherence (proportion of sessions attended), data completeness (proportion of completed survey items and assessments), and acceptability of the intervention (participant-reported satisfaction, perceived appropriateness, and comfort with program components; MultimediaAppendices 1  2).

Secondary objectives include the physical, emotional, and psychological benefits of the Studio’s partner-based yoga programming. This objective aligns with the Studio’s intended program outcomes of improving physical relaxation, enhancing emotional regulation, and fostering greater mind-body awareness among GBTQ participants through touch-focused yoga practices. Corresponding evaluation questions include:

1. How does participation in partner-based yoga impact participants’ physical well-being (eg, flexibility, mobility, and muscle relaxation)?
2. What emotional and psychological benefits do participants report from engaging in touch-focused yoga sessions (eg, stress reduction, self-regulation, and body awareness)?
3. How do these effects vary across different racial or ethnic identities, previous experiences with touch-based movement practices, and LTPA engagement history?

The second objective is to evaluate the role of intentional touch in fostering social connection, trust, and community engagement within the Studio. This objective reflects the Studio’s broader goal of promoting social belonging, interpersonal trust, and normalization of healing touch within GBTQ wellness spaces. Related evaluation questions include:

1. How do participants perceive the role of touch in building trust, social connection, and belonging within the Studio’s yoga classes?
2. Does engagement in these classes increase participants’ comfort with consensual physical contact in LTPA spaces?
3. How does participation in the Studio’s programming influence engagement in other touch-based wellness or movement practices?

## Methods

### Overview

This protocol was developed in accordance with the template for intervention description and replication (TIDieR) guidelines (Checklist 1), ensuring transparent reporting of study design, setting, eligibility criteria, interventions, outcomes, and community involvement [26]. This evaluation will use a mixed methods approach, combining quantitative and qualitative methods to assess the physical, psychological, and social impacts of the Studio’s partner-based, touch-focused yoga classes in New York City. The study will include pre- and postassessments, surveys, interviews, and SNA to evaluate participants’ experiences and the broader impacts of the program.

The partner-based yoga intervention follows a structured yet adaptable format consistent with TIDieR reporting guidelines. The Studio classes are typically 60‐90 minutes in duration and include several core components, such as (1) opening grounding or breathwork exercises to support nervous system regulation; (2) guided partner-based stretching and assisted mobility practices; (3) consent-based touch exercises emphasizing communication, boundary-setting, and participant autonomy; (4) periods of supported relaxation or bodywork-inspired techniques; and (5) optional closing reflections.

Although instructors may vary in style and sequencing, all facilitators are trained in trauma-informed practices, consent-based touch communication, and core program principles emphasizing safety, autonomy, and inclusivity. To promote consistency, instructors follow shared guidelines related to consent practices, class pacing, and participant check-ins, while maintaining flexibility to adapt to group needs and dynamics. Variability in delivery will be documented through observational field notes and coreflection sessions with facilitators to support interpretation of findings.

### Study Design

A convergent parallel design, including a pre-post intervention, will be used at 3 time points—baseline (before observation), immediately post observation, and at a follow-up (4‐6 wk after observation). A flexible 4‐ to 6-week window will be used to balance methodological rigor and feasibility in a community-based setting where attendance patterns may vary; longer-term follow-up assessments (eg, 12 wk) will be considered in future studies once feasibility and retention patterns are established. Accordingly, feasibility and acceptability outcomes are prioritized as primary endpoints, with health-related outcomes examined as exploratory secondary measures.

The pre-post design reflects the exploratory and implementation-focused aims of this protocol and allows for an assessment of both immediate and long-term effects of the program [27]. As the first formal evaluation of a touch-centered partner yoga program in a community-based, GBTQ wellness setting, the study prioritizes feasibility, acceptability, and preliminary outcome patterns over causal inference. A single-group, pre-post design allows for the assessment of within-person change and minimizes disruption to an existing program; whereas, adding a comparison group would introduce logistical challenges (eg, nonparticipation from the Studio) and ethical challenges (eg, depriving a group of potential benefits). Findings will inform future comparative designs once feasibility and implementation processes are established.

Importantly, the absence of a comparison group is intentional and aligned with the feasibility-oriented aims of this protocol. As the first formal evaluation of a touch-centered partner yoga program within a GBTQ community wellness setting, the primary focus is on assessing feasibility, acceptability, and preliminary outcome patterns rather than establishing causal effectiveness. Introducing a comparison group at this stage would pose both logistical and ethical challenges, including withholding access to a potentially beneficial, community-valued intervention and disrupting the naturalistic structure of an existing program.

A single-group pre-post design allows for the assessment of within-person change while preserving ecological validity in a real-world setting. Findings from this evaluation will be used to inform the design of future studies, including the potential development of controlled or quasi-experimental designs to more rigorously assess effectiveness.

### Ethical Considerations

Ethical approval for this study has not yet been obtained. Institutional review board (IRB) submission is planned for September 2026 through the principal investigator’s (JSE) affiliated university, and all study procedures will be reviewed and approved before participant recruitment or data collection. Because this manuscript describes a prospective protocol, approval numbers are not yet available and will be reported in subsequent publications once granted.

This evaluation follows ethical best practices for community-based LGBTQ+ research, with a strong emphasis on confidentiality, awareness of power dynamics, and participant autonomy [28]. All participants will complete an electronic informed consent process before enrollment. The consent form will outline the study purpose, procedures, voluntary participation, potential risks related to touch-based activities, confidentiality protections, compensation, and the right to withdraw at any time without penalty. During baseline orientation, key elements of consent will also be reviewed verbally to ensure comprehension and reinforce trauma-informed practices.

Given the touch-based nature of the intervention, a layered and ongoing consent process will be implemented throughout program participation. In line with established Studio practices and research ethics, participants will be informed that consent is continuous and may be modified or withdrawn at any time without explanation. Facilitators will conduct verbal check-ins before and during partner-based exercises, and participants will be instructed to use clear verbal and nonverbal stop signals to pause or discontinue touch immediately. Potential adverse events include discomfort with touch, triggering of trauma, or withdrawal from sessions; participants may opt out of any exercise without explanation. Facilitators are trained to recognize signs of distress and pause or modify activities accordingly and will receive additional training in trauma-informed communication, boundary-setting, and de-escalation strategies before study implementation. A protocol will be in place for referral to supportive resources (eg, LGBTQ+ counseling services) [28]. Any incidents of discomfort, withdrawal from activities, or boundary concerns will be documented using structured field notes and reviewed during routine evaluation team meetings, as well as monthly with the CAG and Studio leadership, to support participant safety and responsive programming adaptations.

Participant privacy and confidentiality will be protected through the use of deidentified data, secure, encrypted university-managed servers, restricted role-based access to study materials, and removal of identifying details from qualitative transcripts. Pseudonyms will be used in reporting interview data, and SNA outputs will be presented only in aggregate form to prevent identification of individual participants. Survey data will be collected using university-approved Qualtrics, which provides encryption, institutional authentication, and role-based access controls. Only authorized members of the evaluation team will have access to study data. Data will be securely stored under university governance standards and analyzed using RStudio for statistical analyses and Gephi for social network visualization. A transparency framework will engage Studio facilitators, members, and the broader LGBTQ+ wellness community in discussions about findings, including unexpected results, with messaging strategies co-developed to maintain transparency, trust, and alignment with community-centered values.

Recognizing the inherent power dynamics between researchers and participants, a third-party ombudsman (eg, IRB contact) will be available as needed to address grievances and ensure participants feel safe and empowered throughout the evaluation process. Participants will have the opportunity to review their interview transcripts and request modifications to ensure that their experiences are accurately represented.

Finally, although this evaluation focuses on GBTQ men’s engagement with touch-based yoga, findings may inform adaptations for queer women, non-binary individuals, and broader LGBTQ+ communities. A trauma-informed approach will be critical, recognizing that many LGBTQ+ individuals, particularly transgender, nonbinary, Black, Indigenous, and People of Color participants, may experience body dissociation due to systemic and social harm [29].

### Participants

A total of 40‐50 participants will be recruited to support key feasibility objectives, including recruitment, retention, acceptability, and variability in outcomes, while remaining practical within the Studio setting [30]. This range aligns with recommendations for feasibility and pilot studies, where sample sizes are justified based on precision of feasibility parameters rather than statistical power, and commonly fall between 20 and 50 participants [28,29]. A subsample of 15‐20 participants will complete semistructured interviews to provide depth and contextual understanding of participant experiences [31], with this range selected based on evidence that thematic saturation in focused qualitative studies is often achieved within approximately 15‐23 interviews [32]. This combined sample is sufficient to examine preliminary variability in outcomes and inform implementation, rather than detect statistically powered intervention effects. The projected enrollment balances methodological rigor with the size and recruitment capacity of the Studio community, and findings will be used to inform effect size estimation and sample size calculations for future trials.

Inclusion criteria include participants aged 18 years or older, recent (within 12 months) Studio membership, GBTQ participants who self-identify as male, trans male, or gender-diverse, including a male identity, willing to attend at least 1 partner-based, touch-focused yoga class within the study period, and consenting to all study steps. Exclusion criteria include individuals with contraindications for yoga and those who opt out of research participation.

Participants will be recruited from new member registrations, defined as members who have joined the Studio within the past 12 months. We will also use purposive sampling to recruit registered members who have completed fewer than 2 classes to ensure program novelty and diversity in LTPA history, age, and background. Recruitment will occur through the Studio’s membership listservs, class announcements, and social media channels. Eligibility will be screened using an online form, followed by a phone or email confirmation. Flyers within the Studio’s physical location will provide QR codes for sign-up. Recruitment materials will be co-designed with the CAG to ensure inclusive language and affirming representation.

Participants will complete an online consent form before enrollment, which will outline the study purpose, procedures, and rights. The form will include explicit consent for researchers to access the Studio attendance records to verify participation. Consent will be confirmed electronically, and participants will receive a copy for their records. Participants may pause or withdraw at any time, and facilitators may modify or halt activities if distress or discomfort occurs. During baseline orientation, the research team will review key points verbally, emphasizing voluntary participation, the option to withdraw at any time, and the ability to decline specific activities or questions. Harms will be monitored nonsystematically through facilitator observation and participant self-report.

Participants will receive US $25 gift cards for completing each survey (baseline, postintervention, and follow-up), up to US $75 total, and an additional US $25 gift card for participation in interviews. To support retention across study time points, participants will receive reminders before each survey, timely delivery of incentives, flexible scheduling, affirming follow-up messages, and consistent updates on study progress.

At this time, study materials will be administered in English only due to resource constraints; language accessibility and translation strategies will be explored in future iterations to expand inclusivity.

### Measures

Data will be gathered at 3 time points—baseline assessments (physical, psychological, and social) will be conducted before participants’ first class; during the study period, postobservation assessments will be completed immediately after the program ends; and follow-up assessments will occur 4‐6 weeks later to evaluate short-term maintained effects. Quantitative and qualitative data will be collected in parallel, allowing for a shorter study time frame and richer information while ensuring the rigor necessary for the credibility of overall conclusions [31]. Detailed documentation and study measures are available in the Multimedia Appendix 1. These materials provide the purpose, equipment, procedure, items (where applicable), and scoring guidelines for each instrument—the Sit-and-Reach Test, Goniometry for Joint Flexibility, the PSS-10, the semistructured interview guide, and the Comfort, Connection, and Touch Survey (LGBTQ+-Affirming Adaptation).

The Comfort, Connection, and Touch Survey (LGBTQ+-Affirming Adaptation) is an exploratory instrument developed for this evaluation to capture culturally specific experiences related to consent, comfort with touch, and relational dynamics. Because this survey has not undergone formal psychometric validation, results will be interpreted descriptively. Pilot testing with CAG members and Studio facilitators will be used to assess clarity and feasibility, and findings will inform future refinement and validation of the measure.

Additionally, outcomes are prespecified to align with the feasibility-oriented aims of this protocol. Primary outcomes focus on feasibility and acceptability indicators, including recruitment rate (proportion of eligible participants enrolled), retention rate (proportion completing all study time points), attendance or adherence (proportion of sessions attended), data completeness (proportion of completed survey items and assessments), and acceptability of the intervention (participant-reported satisfaction, perceived appropriateness, and comfort with program components).

Exploratory secondary outcomes include preliminary physical, psychological, and social indicators, including perceived stress (PSS-10), body awareness (MAIA), resilience (BRS), physical flexibility and mobility (sit-and-reach and goniometry), and social connectedness assessed through SNA (eg, network density and centrality). Qualitative indicators of comfort with touch, emotional regulation, and belonging will also be examined. These outcomes are exploratory and intended to inform future hypothesis-driven trials.

To support the interpretation of feasibility and inform decisions regarding scale-up, a priori thresholds will be applied. Feasibility will be considered acceptable if recruitment reaches ≥70% of eligible participants, retention is ≥80% across all time points, attendance or adherence is ≥75% of scheduled sessions, and data completeness is ≥85% across measures. Acceptability will be considered high if mean participant satisfaction scores are ≥4 out of 5. This is consistent with early-stage feasibility and pilot intervention research, where recruitment, retention, adherence, and participant acceptability are emphasized as key indicators of implementation success and progression to future trials [28,29]. These criteria will guide decisions regarding program refinement, potential scale-up, and progression to future controlled trials.

### Quantitative Measures

The study will assess physical, psychological, and social outcomes related to the Studio’s partner-based, touch-focused yoga classes. Physical outcomes will be measured through pre- and postflexibility assessments, including the Sit-and-Reach Test, which evaluates lower back and hamstring flexibility, and goniometry, which assesses joint mobility of the hip flexors, hamstrings, and shoulders. Additionally, participants’ baseline physical activity levels and yoga engagement will be measured using a modified version of the International Physical Activity Questionnaire, which captures self-reported activity levels [33].

Psychological outcomes will be assessed using the PSS-10, a validated 10-item instrument that measures stress levels; the MAIA, a tool that evaluates body awareness, emotional regulation, and mind-body awareness; and the emotional resilience and coping mechanisms will be evaluated using a modified version of the BRS [6,34,35].

Social outcomes will be examined through SNA, to track the depth of their relationships within the community and to assess how these connections evolve [36]. SNA will analyze network density, centrality, and community cohesion, helping to evaluate the Studio’s role in fostering a connected queer wellness community. Network nodes will consist of consenting study participants only; Studio instructors, staff, and nonparticipating members will not be included as nodes to preserve consistency in network boundaries and protect confidentiality. Network ties will be defined a priori based on participant self-report and will include indicators, such as (1) participation in shared partner-based yoga activities, (2) perceived emotional or social support, and (3) self-identified meaningful connections within the Studio. Participants will complete social network survey items at each time point to identify connections with other participants. These data will be used to construct network matrices and visualize changes in relational structure over time. Key network metrics will include density (overall connectedness of the network), degree centrality (number of connections per participant), and clustering coefficients (subgroup cohesion). These metrics will be used to assess changes in social integration, trust-building, and community cohesion associated with program participation.

### Qualitative Measures

Data will also be collected through semistructured interviews with a diverse selection of 15‐20 participants, or when saturation is reached, conducted after the observational period [31]. These interviews will explore participants’ experiences with touch, including comfort, consent, and emotional response, as well as the perceived psychological and social benefits of partner-based yoga. Barriers and facilitators to engagement in touch-focused yoga will also be explored. A trauma-informed approach will guide the interview process, with question guides co-developed with the CAG to ensure cultural relevance and emotional safety. Open and closed-ended survey questions will be included in postobservation surveys to capture participants’ reflections on emotional and physical changes, their comfort with touch, and their sense of belonging within the Studio community.

### Data Analysis Plan

Data will be entered into and stored on encrypted university servers. Basic data quality procedures will include range checks during data entry, automated validation rules within survey platforms, and periodic verification of exported datasets to identify inconsistencies or missing values before analysis.

All enrolled, consenting participants with baseline data will be included in the analysis. Feasibility outcomes will be analyzed using descriptive statistics (eg, proportions, means, and SDs), with no hypothesis testing for primary feasibility indicators. Quantitative data will be analyzed using descriptive statistics (means and SDs) to summarize all pre- and postintervention measures. Missing values will be handled with pairwise deletion and sensitivity checks. Paired *t* tests for parametric data and Wilcoxon signed-rank tests for nonparametric data will be used to compare changes in flexibility, stress, body awareness, and resilience. Repeated-measures ANOVA will be used to assess statistically significant differences across one or more time points (baseline, postintervention, and follow-up). SNA conducted in Gephi will illustrate network density, degree centrality, and clustering coefficients to quantify changes in social connections over time. Network boundaries will include only consenting participants, and ties will be defined a priori as self-reported interaction, support, or shared partner-based activities. Individuals who do not consent will be excluded from network datasets. Prespecified metrics include density and centrality aligned with social connectedness outcomes. To reduce reidentification risk, network outputs will be anonymized and aggregated, and synthetic network examples may be used for open-science sharing rather than raw network structures. Longitudinal analysis will track shifts in community-building, with changes in network density and centrality directly addressing evaluation questions related to social connectedness, trust-building, and participants’ sense of belonging, offering measurable indicators of the Studio’s community impact.

To support the interpretation of the outcomes, potential moderators and contextual variables will be explored descriptively, including previous experience with touch-based practices, mental health support context, and trauma-related considerations, where feasible. Because this protocol prioritizes feasibility, these variables are not powered for formal subgroup analyses but will inform future study considerations.

Qualitative data will be examined through thematic analysis of semistructured interview transcripts using NVivo 12 software (Lumivero). This analysis will explore participants’ experiences with touch, emotional responses, and perceived social benefits, as well as barriers and facilitators to engagement. Thematic coding, developed from transcriptions and field notes, will also address issues of consent, safety, and trauma-informed practices [37]. Furthermore, 2 coders (JSE and PER) will use criteria adapted from the study by Bowen [38] to identify categories that have been adequately saturated, including categories reflecting more than 70% of the interviews. Finally, qualitative and quantitative findings will be integrated through mixed methods triangulation. A joint display matrix will be used to align emerging qualitative themes with meaningful quantitative differences, enabling the identification of convergent or divergent patterns across data sources [31,39]. Data visualization illustrating results from the PSS-10 and a social network map visualizing participant relationships before and after the observation period will be included in the final report. Deidentified data, code, and instruments will be shared via an open-access repository after publication with a data dictionary and codebook.

### Dissemination Plan

Findings from this evaluation will be disseminated through a multipronged approach designed to ensure accessibility, engagement, and meaningful application for the Studio facilitators, participants, and wellness-based LGBTQ+ organizations in and beyond New York City. Primary dissemination channels will include internal reporting to Studio leadership and facilitators, community-focused communications, and academic and practitioner-oriented publications.

Internally, a comprehensive executive summary will be developed, highlighting key findings related to participant outcomes, social network dynamics, and program effectiveness. This report will include data visualizations, thematic analysis with supporting excerpts, and actionable recommendations for the Studio facilitators to refine programming and enhance participant experiences.

For community engagement, an infographic summarizing the most significant results will be distributed through the Studio’s email newsletters, website, and social media platforms, ensuring research transparency, accessibility for current and prospective members, and responsible marketing development. Accordingly, focused-impact social media content (eg, Instagram posts and short-form video summaries) will be co-developed with the Studio facilitators to present findings in an engaging and digestible manner. LGBTQ+ wellness organizations and advocacy groups in New York City will also receive tailored presentations to foster dialogue on best practices for integrating healing touch in community wellness spaces.

For academic and professional dissemination, findings will be shared at public health and LGBTQ-centered conferences, with manuscripts submitted to peer-reviewed journals in behavioral health, trauma-informed care, and somatic healing spaces. Additionally, policy briefs will be drafted for organizations involved in LGBTQ+ health and wellness advocacy, positioning the Studio’s program as a case study for trauma-informed, community-based physical activity interventions.

### Evaluation Team and Timeline

The proposed evaluation team and timeline (Table 1) are structured to balance expertise in research methods, community engagement, and data analysis, ensuring both rigor and cultural responsiveness. The team will consist of the lead evaluator (JSE; 40% time; 832 h/y; 16 h/wk for 52 wk), responsible for evaluation design, community engagement, data interpretation, and final report development; data analyst (30% time; 624 h/y; 12 h/wk for 52 wk), who oversees quantitative data management, statistical analyses, and data visualization using NVivo 12 and R Studio; and community liaison (30% time; 624 h/y; 12 h/wk for 52 wk), who facilitates CAG and leadership meetings and semistructured interviews, ensuring the evaluation process remains participant-centered and trauma-informed.

**Table 1.** Evaluation timeline with activities, phases, and team roles.

Activity	Timeline	Responsible team members
IRB^a^ submission and instrument finalization	September 2026-July 2027	Lead evaluator^b^ (design and oversight), community liaison (CAG^c^ coordination)
Recruitment of participants	July 2027	Lead evaluator (strategy), community liaison (member outreach and screening)
Baseline data collection	July 2027 until completion	Data analyst (survey setup and data capture), community liaison (interviews and attendance verification)
Intervention period and postintervention data collection	July 2027 until completion	Lead evaluator (implementation oversight), data analyst (data management), community liaison (facilitator coreflection)
Follow-up data collection (4‐6 wk)	March 2027	Data analyst (survey administration and monitoring), community liaison (participant retention support)
Data analysis	September 2027-May 2028	Data analyst (statistical analysis, SNA^d^, and visualization), lead evaluator (interpretation and integration)
Dissemination (reports, community presentations, and manuscripts)	May 2028 onward	Lead evaluator (final reporting and manuscripts), community liaison (community presentations), data analyst (visualizations and figures)
Weekly coordination meetings	Ongoing throughout project	Entire evaluation team (continuous alignment and troubleshooting)

a IRB: institutional review board.

b Lead evaluator: JSE.

c CAG: community advisory group.

d SNA: social network analysis.

Weekly 60-minute evaluation team meetings (via Zoom [Zoom Communications, Inc]) will ensure coordination across data collection, analysis, and community engagement activities, allowing for continuous troubleshooting and alignment of evaluation tasks.

## Results

As of March 2026, this study has not yet begun. IRB submission is planned for September 2026. Following approval, study instruments will undergo a brief pilot testing and feasibility assessment phase with Studio facilitators and CAG members to evaluate clarity, cultural responsiveness, and implementation logistics before initiating full participant recruitment. Participant recruitment will ideally begin in July 2027, with data collection continuing through late 2027. Data analysis and dissemination of findings are expected in 2028.

## Discussion

### Principal Findings

This protocol outlines a mixed methods evaluation of the Studio’s touch-based yoga programming for GBTQ men in New York City. By centering consensual touch within LTPA, this project addresses a significant gap in both public health research and LGBTQ+ wellness practice, intervention, methodology, and research. Previous studies have demonstrated the psychosocial and physiological benefits of partner-based yoga in clinical and nonclinical populations, including improvements in stress, interpersonal connection, and emotional well-being [40,41]. However, few studies have examined these practices in LGBTQ+ communities, where opportunities for affirming touch and safer embodiment are limited [42,43], and the benefits of intentional touch may mitigate queer disparities in depression, anxiety, and loneliness. This protocol, therefore, represents a novel contribution by adapting evidence-based methods of evaluation to a culturally specific, community-driven intervention.

### Significance and Contribution to Previous Research

This evaluation builds upon growing evidence that intentional touch in physical activity settings can reduce stress, enhance body awareness, and strengthen social cohesion [6,35]. It expands upon earlier partner yoga and touch-based intervention studies by situating the work within a wellness community designed by and for GBTQ men. In doing so, the project responds to calls for more inclusive and culturally responsive approaches to physical activity research [23,44], while also modeling how trauma-informed and equity-centered frameworks can be integrated into program evaluation [45]. Findings from this work will have implications not only for the Studio’s programming but also for broader efforts to normalize healing touch in LGBTQ+ wellness spaces and beyond.

### Strengths and Limitations

The strengths of this protocol include its participatory design, which embeds community members in all stages of instrument development, data collection, and interpretation through a CAG [24,25]. This approach increases cultural validity and ensures that findings are meaningful to those most directly impacted. The mixed methods design—combining flexibility and stress measures, validated psychosocial scales, and SNA with in-depth interviews—also provides a comprehensive approach to assessing several dimensions of program outcomes [31,39].

However, the study has limitations. Importantly, recruitment is limited to individuals who have voluntarily enrolled in the Studio, a touch-centered and queer-affirming wellness environment. As a result, participants are likely to have a higher baseline affinity for interpersonal touch, embodied practices, and LGBTQ+-inclusive spaces compared with the broader GBTQ population. This self-selection may introduce selection bias, potentially limiting generalizability and attenuating observed pre-post changes due to ceiling effects in psychosocial outcomes, such as comfort with touch, belonging, or emotional regulation. In this context, smaller observed changes should not be interpreted as a lack of program effectiveness but rather as reflecting a population that may already be partially aligned with the intervention’s core components. Additionally, findings may not generalize to individuals who experience discomfort with touch, have histories of trauma related to physical contact, or lack access to affirming wellness spaces. Future studies should include more diverse recruitment strategies and comparative designs to assess how outcomes differ across populations with varying baseline levels of touch exposure and comfort.

All the Studio classes are currently taught in English, which excludes non–English-speaking participants. Additionally, while the evaluation incorporates trauma-informed practices, some individuals may still experience discomfort with partner-based or touch-focused activities, which could influence both participation and reported outcomes.

### Future Considerations

Future iterations of this work should expand programming to include trauma-informed modifications, such as consent-based touch choices and self-touch practices. Enhancing inclusivity also requires the integration of gender-neutral language, avoidance of gendered movement assumptions, and consistent use of pronoun-sharing practices. Facilitators should receive ongoing education on intersectional LGBTQ+ identities to strengthen inclusivity and responsiveness [44]. Furthermore, access to affirming and celebratory wellness spaces must remain central, with particular attention to extending healing touch practices to queer women, nonbinary individuals, transgender women, and other gender-diverse participants. Subsequent evaluations should consider offering programming and instruments in Spanish and other languages to expand accessibility. Finally, spiritual development, a primary feature of the Studio’s programming, warrants exploration in future research to capture the full scope of participant experiences.

## Supplementary material

10.2196/86310 Multimedia Appendix 1 Quantitative measures survey.

10.2196/86310 Multimedia Appendix 2 Interview guide.

10.2196/86310 Checklist 1 TIDieR checklist.

## Abbreviations

BRS: Brief Resilience Scale
CAG: community advisory group
GBTQ: gay, bisexual, transgender, and queer
IRB: Institutional review board
LGBTQ+: lesbian, gay, bisexual, transgender, questioning, or queer and others
LTPA: leisure-time physical activity
MAIA: Multidimensional Assessment of Interoceptive Awareness
PSS-10: Perceived Stress Scale-10 items
SNA: social network analysis
TIDieR: template for intervention description and replication
