# Supplementary material for: Touch-Based Partner Yoga for Gay, Bisexual, Transgender, and Queer Men in a Community Wellness Setting: Protocol for a Mixed Methods Program Evaluation of “The Studio”
Source: JMIR Res Protoc. 2026 Apr 29;15:e86310. doi: 10.2196/86310 (PMC13128061; doi:10.2196/86310)
Supplement: Multimedia Appendix 1 [file resprot-v15-e86310-s001.docx]

**Quantitative Measures Survey**

This survey integrates all quantitative measures into one instrument. Some sections require physical assessments conducted by facilitators (Sit-and-Reach, Goniometry). Other sections are self-report scales completed directly by participants (PSS-10, MAIA, BRS, SNA, IPAQ).

# Section 1: Physical Tests

**1. Sit-and-Reach Test**

Instructions: Sit with legs extended, feet against the box/ruler. Reach forward with both hands, keeping knees straight. Record the farthest reach (cm/inches). Best of three attempts recorded.

| **Attempt** | **Distance (cm/inches)** |
| --- | --- |
| Attempt 1 |  |
| Attempt 2 |  |
| Attempt 3 |  |

**2. Goniometry for Joint Flexibility**

Instructions: Measure degrees of movement using a goniometer. Record results below.

| **Movement** | **Degrees** |
| --- | --- |
| Hip Flexion |  |
| Hamstring Extension |  |
| Shoulder Flexion |  |

#

# Section 2: Self-Report Scales

**3. Perceived Stress Scale (PSS-10)**

Response scale: 0 = Never, 1 = Almost Never, 2 = Sometimes, 3 = Fairly Often, 4 = Very Often

1. In the last month, how often have you been upset because something that happened unexpectedly?
2. In the last month, how often have you felt that you were unable to control the important things in your life?
3. In the last month, how often have you felt nervous and “stressed”?
4. In the last month, how often have you felt confident about your ability to handle personal problems? (Reversed)
5. In the last month, how often have you felt that things were going your way? (Reversed)
6. In the last month, how often have you found that you could not cope with all the things that you had to do?
7. In the last month, how often have you been able to control irritations in your life? (Reversed)
8. In the last month, how often have you felt that you were on top of things? (Reversed)
9. In the last month, how often have you been angered because of things outside of your control?
10. In the last month, how often have you felt difficulties were piling up so high that you could not overcome them?

**4. Multidimensional Assessment of Interoceptive Awareness (MAIA)**

Response scale: 0 = Never, 1 = Rarely, 2 = Sometimes, 3 = Often, 4 = Always

1. When I am tense I notice where the tension is located in my body. (Noticing)
2. I notice when I am uncomfortable in my body. (Noticing)
3. I notice where in my body I am comfortable. (Noticing)
4. I notice changes in my breathing, such as whether it slows down or speeds up. (Noticing)
5. I am aware of changes in my heartbeat. (Noticing)
6. 6. I ignore physical tension or discomfort until they become more severe. (Not-Distracting, reversed)
7. I distract myself from sensations of discomfort. (Not-Distracting, reversed)
8. When I feel pain or discomfort, I try to ignore it. (Not-Distracting, reversed)
9. I start to worry that something is wrong if I feel any discomfort. (Not-Worrying, reversed)
10. I can notice an unpleasant body sensation without worrying about it. (Not-Worrying)
11. When I feel pain in my body, I become upset. (Not-Worrying, reversed)
12. I can pay attention to my breath without being distracted by things happening around me. (Attention Regulation)
13. I can maintain awareness of my inner bodily sensations even when there is a lot going on around me. (Attention Regulation)
14. I can refocus my attention on my body if I am distracted. (Attention Regulation)
15. I can sustain attention to my bodily sensations for long periods of time. (Attention Regulation)
16. I notice how my body changes when I am angry. (Emotional Awareness)
17. When something is wrong in my life I can feel it in my body. (Emotional Awareness)
18. I notice that my breathing becomes free and easy when I feel comfortable. (Emotional Awareness)
19. I notice how my body feels when I am happy. (Emotional Awareness)
20. When I am sad I notice where I can feel this in my body. (Emotional Awareness)
21. I listen to my body to inform me about what to do. (Body Listening)
22. When I am upset, I take time to explore how my body feels. (Body Listening)
23. I listen for information from my body about my emotional state. (Body Listening)
24. When I am tense I take notice of where the tension is in my body. (Body Listening)
25. When I feel overwhelmed I can find a calm place inside. (Self-Regulation)
26. I can use my breath to reduce tension. (Self-Regulation)
27. When I am caught up in thoughts, I can calm my mind by focusing on my body/breath. (Self-Regulation)
28. I trust my body sensations. (Trusting)
29. I am at home in my body. (Trusting)
30. I feel my body is a safe place. (Trusting)
31. I trust that my body can give me information about my well-being. (Trusting)
32. I feel my body helps me to cope with difficult situations. (Trusting)

**5. Brief Resilience Scale (BRS)**

Response scale: 1 = Strongly Disagree, 2 = Disagree, 3 = Neutral, 4 = Agree, 5 = Strongly Agree

1. I tend to bounce back quickly after hard times.
2. I have a hard time making it through stressful events. (Reversed)
3. It does not take me long to recover from a stressful event.
4. It is hard for me to snap back when something bad happens. (Reversed)
5. I usually come through difficult times with little trouble.
6. I tend to take a long time to get over set-backs in my life. (Reversed)

**6. Social Network Analysis (SNA)**

Instructions: Please list up to 10 participants you interact with most in this program. For each, indicate:

- Frequency of contact (Daily / Weekly / Monthly)
- Strength of relationship (1–5)
- Type of interaction (In-person / Online / Both)

**7. International Physical Activity Questionnaire (IPAQ, Short Form)**

Instructions: Answer questions about your physical activity over the last 7 days.

1. During the last 7 days, on how many days did you do vigorous physical activities like heavy lifting, aerobics, or fast bicycling?
2. How much time did you usually spend on one of those days doing vigorous physical activities?
3. During the last 7 days, on how many days did you do moderate physical activities like carrying light loads, bicycling at a regular pace, or doubles tennis?
4. How much time did you usually spend on one of those days doing moderate physical activities?
5. During the last 7 days, on how many days did you walk for at least 10 minutes at a time?
6. How much time did you usually spend on one of those days walking?
7. During the last 7 days, how much time did you usually spend sitting on a weekday?

**8. Comfort, Connection, and Touch Survey (LGBTQ+-Affirming Adaptation)**

**Purpose**

To assess participants’ comfort, connection, and perceptions of touch in the Studio yoga classes.

**Instructions**

Rate your agreement with each statement on a scale from 1 (Strongly Disagree) to 5 (Strongly Agree).

**Survey Items**

1. I felt comfortable with the amount of touch used during class.
2. The facilitator clearly explained consent practices related to touch.
3. I felt respected in my personal boundaries around touch.
4. Touch in class enhanced my sense of physical comfort.
5. Touch in class enhanced my sense of emotional comfort.
6. Touch in class increased my feelings of connection to other participants.
7. I felt a stronger sense of belonging within the the Studio community after class.
8. Touch during class supported my ability to relax and release stress.
9. I felt more aware of my body and breath because of touch.
10. I would feel comfortable recommending touch-based classes at the Studio to others.

**Optional Open-Ended Questions**

- Please describe a moment in class when touch felt particularly meaningful to you.
- Were there any moments when touch felt uncomfortable or unclear?
- What suggestions do you have for making touch-based classes more affirming and inclusive?

**Scoring**

Responses to Likert-scale items will be averaged to generate sub-scores for comfort, connection, and belonging. Open-ended responses will be analyzed thematically to capture nuanced experiences of touch.

**References**

1. Craig CL, Marshall AL, Sjöström M, et al. International physical activity questionnaire: 12-country reliability and validity. *Med Sci Sports Exerc.* 2003;35(8):1381–1395. doi:10.1249/01.Mss.0000078924.61453.Fb
2. Cohen S, Kamarck T, Mermelstein R. A global measure of perceived stress. *J Health Soc Behav.* 1983;24(4):385–396.
3. Mehling WE, Price C, Daubenmier JJ, Acree M, Bartmess E, Stewart A. The Multidimensional Assessment of Interoceptive Awareness (MAIA). *PLoS One.* 2012;7(11):e48230. doi:10.1371/journal.pone.0048230
4. Smith BW, Dalen J, Wiggins K, Tooley E, Christopher P, Bernard J. The brief resilience scale: assessing the ability to bounce back. *Int J Behav Med.* 2008;15(3):194–200.
5. Grewal E, Godley J, Wheeler J, Tang KL. Use of social network analysis in health research: a scoping review protocol. *BMJ Open.* 2024;14(5):e078872. doi:10.1136/bmjopen-2023-078872
