# Supplementary material for: Touch-Based Partner Yoga for Gay, Bisexual, Transgender, and Queer Men in a Community Wellness Setting: Protocol for a Mixed Methods Program Evaluation of “The Studio”
Source: JMIR Res Protoc. 2026 Apr 29;15:e86310. doi: 10.2196/86310 (PMC13128061; doi:10.2196/86310)
Supplement: Multimedia Appendix 2 [file resprot-v15-e86310-s002.docx]

**Semi-Structured Interview Guide**

**Purpose**

To explore participants’ experiences with touch in The Studio’s yoga programming, including comfort, consent, connection, and perceived benefits.

**Introduction**

- Welcome and thanks: “Thank you for taking the time to talk with me today.”
- Purpose: “The goal of this conversation is to learn about your experiences in the Studio’s touch-based yoga classes. We’re especially interested in how touch feels in these settings and what it means for your well-being and sense of community.”
- Consent reminder: “You’ve already given consent, but I’d like to remind you that your participation is voluntary. You can skip any question or stop the interview at any time. Everything you share will be kept confidential.”
- Check-in: “Do you have any questions before we begin?”

**Warm-Up Questions**

- Can you tell me about your prior yoga experience?
  - Prompt if needed: “For example, have you practiced yoga before, or is the Studio your first experience?”
- What interested you in trying partner-based or touch-focused yoga?
  - Prompt if needed: “Was it curiosity, recommendations, community connection, or something else?”

**Core Questions**

1. **Experiences with touch**
   1. “How would you describe your experiences with touch in the Studio classes?”
   2. Prompt: “Were there moments when touch felt affirming, challenging, or surprising?”
2. **Memorable moment**
   1. “Can you share a memorable or impactful moment involving touch during a class?”
   2. Prompt: “It could be positive, challenging, or something that stood out for you.”
3. **Comfort and consent**
   1. “How has touch in these classes influenced your comfort with physical contact?”
   2. Prompt: “Do you feel more, less, or about the same comfort compared to before?”
4. **Emotional impact**
   1. “What effect, if any, has touch had on your emotional well-being?”
   2. Prompt: “Has it affected your stress levels, mood, or sense of relaxation?”
5. **Community connection**
   1. “How has participating in these classes shaped your sense of community at the Studio?”
   2. Prompt: “Do you feel more connected to others? In what ways?”
6. **Outside relationships**
   1. “Have your experiences with touch in the Studio influenced your relationships or interactions outside of class?”
   2. Prompt: “For example, comfort with friends, partners, or other wellness settings.”
7. **Suggestions for improvement**
   1. “What suggestions do you have for improving the touch-based yoga experience at the Studio?”
   2. Prompt: “This could relate to class structure, facilitator guidance, or how consent is addressed.”

**Considerations**

- Use gender-neutral and inclusive language.
- Allow participants to skip questions at any time.
- Validate experiences and emphasize participant control over what they share.
- Employ a trauma-informed approach throughout.

**Scoring / Analysis**

Interviews will be audio-recorded (with permission), transcribed, and analyzed using thematic analysis (Braun & Clarke, 2006). Codes and themes will focus on comfort, consent, emotional impact, and community connection.

**References**

Braun V, Clarke V. Using thematic analysis in psychology. *Qualitative Research in Psychology*. 2006/01 2006;3(2):77-101. doi:10.1191/1478088706qp063oa
