# Supplementary material for: Touch-Based Partner Yoga for Gay, Bisexual, Transgender, and Queer Men in a Community Wellness Setting: Protocol for a Mixed Methods Program Evaluation of “The Studio”
Source: JMIR Res Protoc. 2026 Apr 29;15:e86310. doi: 10.2196/86310 (PMC13128061; doi:10.2196/86310)
Supplement: Checklist 1 [file resprot-v15-e86310-s003.pdf]

TIDieR

The Studio: a community-based, touch-centered partner yoga program for gay, bisexual, transgender, and queer (GBTQ) men in a membership-based wellness setting in New York City, evaluated via a mixed-methods program evaluation protocol.

Why:

The Studio's touch-centered partner yoga programming is designed to address two related gaps: (1) limited access to affirming, inclusive leisure-time physical activity (LTPA) spaces for GBTQ men and (2) limited access to safe, consensual, non-sexual healing touch within structured movement settings. The intervention assumes that combining structured movement with intentional, consent-based touch may produce synergistic benefits across physical, psychological, and social domains. Touch is treated as an essential "active ingredient" rather than an incidental feature because it is intentionally integrated to foster interpersonal trust, belonging, and normalization of healing touch in GBTQ wellness spaces. Consent functions as a core mechanism of action, not only an ethical safeguard. Ongoing consent and boundary negotiation are expected to strengthen autonomy and psychological safety, which may increase comfort with touch-based movement, support self-regulation, and improve engagement in embodied practices.

This protocol is a feasibility-oriented, mixed-methods program evaluation of an existing community-based intervention. The evaluation goals are to (1) assess individual outcomes associated with participation in partner-based, touch-focused yoga (physical, emotional, psychological) and (2) examine interpersonal and community outcomes of intentional touch within the Studio context (social connection, trust, belonging), using pre/post and short-term follow-up assessments while minimizing disruption to a real-world program.

What (material):

Program and setting materials

• The Studio's in-person membership-based wellness space in New York City (NYC) used for private, member-only partner-based and touch-focused yoga classes.
• Standard yoga studio infrastructure used in typical partner yoga classes (for example, mats and props as used by the Studio). Specific prop brands and models are not specified in the protocol.

Intervention content materials (as described in the program overview)
• Structured partner-based yoga class content emphasizing intentional, consent-based touch integrated into partner practices.
• Related Studio programming that may occur within the broader organization (for example, bodywork education such as massage, breathwork and self-regulation practices, energy healing, spiritual rituals, and immersive events, plus an online community platform). The evaluation described in this protocol focuses on the touch-based yoga classes delivered in NYC.

Evaluation and implementation support materials
• Electronic informed consent materials, including explicit consent to access Studio attendance records for participation verification.
• Trauma-informed participation supports used during classes, including ongoing consent prompts, facilitator check-ins, and clear verbal and nonverbal stop signals to pause or discontinue touch.

• Data collection platforms and instruments: UNC Charlotte–approved Qualtrics surveys; a social connectedness and social network survey component used for social network analysis (SNA); a semi-structured interview guide co-developed with the Community Advisory Group (CAG); and the exploratory Comfort, Connection, and Touch Survey (LGBTQ+-Affirming Adaptation).
• Physical assessment equipment and documentation: sit-and-reach test materials and goniometry materials for joint flexibility and mobility assessment (purpose, equipment, procedures, and scoring are described as available in supplementary materials).
• Analysis software: RStudio for quantitative analysis; Gephi for social network visualization; NVivo 12 for qualitative thematic analysis.

Where materials can be accessed
• The protocol indicates that detailed documentation and study measures (including the sit-and-reach test, goniometry procedures, PSS-10, interview guide, and the Comfort/Connection/Touch survey adaptation) are provided in the supplementary materials. Materials are administered in English only at this time.

What (procedures):

Intervention procedures (Studio programming being evaluated)

• Participants take part in The Studio's existing partner-based, touch-focused yoga classes delivered as private, member-only sessions at the NYC location. Classes emphasize intentional, consent-based touch integrated into partner yoga practices to support relaxation, muscle relief, embodied connection, and community building.

Consent and safety procedures embedded in delivery
• Participants complete electronic informed consent prior to enrollment, with key points reviewed verbally during baseline orientation to reinforce trauma-informed practices.
• Consent is ongoing throughout participation and may be modified or withdrawn at any time without explanation.
• Facilitators conduct brief verbal check-ins before and during partner-based exercises.
• Participants use clear verbal and nonverbal stop signals to pause or discontinue touch immediately.
• Participants may opt out of any exercise without explanation.
• Facilitators pause, modify, or halt activities if distress or discomfort occurs.
• Referral protocol for supportive resources (for example, LGBTQ+ counseling services) is available as needed.
• Incidents of discomfort, withdrawal, or boundary concerns are documented using structured field notes and reviewed during routine evaluation team meetings and monthly with the CAG and Studio leadership to support responsive programming adaptations.

Evaluation procedures that structure implementation and measurement
Recruitment and eligibility
• Recruit approximately 40–50 participants from new Studio members (joined within the past 12 months), with purposive recruitment of members who have completed fewer than two classes to support novelty and diversity in LTPA history and background.
• Recruitment via membership listservs, class announcements, social media, and flyers or QR codes in the physical location. Eligibility screening is completed online with follow-up confirmation by phone or email. Recruitment materials are co-designed with the CAG.

Assessment schedule (three time points)
• Baseline (pre-observation) assessments occur before the participant's first class during the study period.
• Post-observation assessments occur immediately after the observation period ends.
• Follow-up assessments occur 4–6 weeks post-observation to evaluate short-term sustained effects.

Quantitative procedures
• Physical assessments: sit-and-reach and goniometry for flexibility and mobility (hip flexors, hamstrings, shoulders) administered pre and post.

• Surveys: Perceived Stress Scale (PSS-10); selected MAIA subscales; Brief Resilience Scale (modified); modified IPAQ; and SNA measures to assess social connectedness and network structure.

Qualitative procedures
• Semi-structured interviews with approximately 15–20 participants (or until saturation) conducted after the observation period using a trauma-informed approach. Interview guides are co-developed with the CAG.

Community partnership and reflection procedures
• The CAG (5–10 Studio members who are not study participants) co-develops and approves evaluation administration, provides input on instruments, and contributes to interpretation of results.
• Biweekly co-reflection sessions with Studio facilitators and leadership review study progress and embed real-time reflection into evaluation and refinement processes.

Who provided:

Intervention providers

• The Studio's partner-based, touch-focused yoga classes are delivered by Studio facilitators and yoga instructors within the NYC membership-based wellness setting. The protocol characterizes providers as certified yoga instructors with training relevant to bodywork-informed approaches and trauma-informed practices.

Training relevant to delivering a touch-based intervention safely
• Facilitators will receive training in trauma-informed communication, boundary-setting, and de-escalation strategies prior to study implementation to support ongoing consent processes and appropriate responses to distress during partner-based exercises.

Personnel involved in evaluation (not intervention delivery)
• The evaluation is implemented by a research and evaluation team with defined roles (Lead Evaluator, Data Analyst, Community Liaison) responsible for instrument development with the CAG, survey administration, data management and analysis, facilitation of CAG and leadership meetings, interviews, attendance verification, and dissemination activities.

How (mode of delivery; individual or group):

The intervention is delivered primarily through in-person, face-to-face classes at The Studio's NYC location. Delivery is interactive and relational, requiring real-time partner engagement and consent negotiation during touch-based exercises. The intervention occurs in a group class context, with partner-based activities embedded within sessions. Participants engage in dyads or partner configurations during class. Specific group size parameters and partner rotation rules are not specified in the protocol. The broader Studio ecosystem includes additional programming and an online community platform, but the evaluation described in this protocol focuses on the touch-based yoga classes delivered in NYC.

Where:

The intervention occurs at The Studio's membership-based wellness space in New York City (NYC), delivered through private, member-only partner-based and touch-focused yoga classes. The setting is a real-world community program rather than a clinical environment and includes membership infrastructure (for example, listservs), on-site recruitment capacity (flyers or QR codes), and attendance records that can be accessed with participant consent to verify participation. The broader organization includes both in-person and online programming, but the primary evaluation setting is the NYC physical location.

When and how much:

Planned timing and exposure in a real-world community program

• The Studio's partner-based, touch-focused yoga classes are ongoing offerings within the NYC membership-based program. This protocol evaluates participation in an existing community-based intervention with real-world variability in attendance patterns. Specific class frequency, session length, and a fixed curriculum dose are not specified in the protocol.

Minimum exposure requirement

• Eligibility requires willingness to attend at least one partner-based, touch-focused yoga class within the study period.

Assessment schedule
• Baseline assessments are conducted prior to the participant’s first class during the study period.
• Post-observation assessments are completed immediately after the observation period ends.
• Follow-up assessments occur 4–6 weeks post-observation. The follow-up window is flexible (4–6 weeks) to accommodate attendance patterns and feasibility.

How dose will be captured
• Actual intervention dose will be quantified using Studio attendance records (with explicit participant consent) and reported as attendance and retention metrics (for example, number of classes attended during the observation period), enabling analysis of outcomes in relation to real-world participation level.

Tailoring:

Yes. The intervention is planned to be adapted in real time through a consent-centered, trauma-informed model in which participants do not receive identical touch exposure or partner-based activities. Tailoring occurs through: ongoing consent that may be modified or withdrawn at any time; facilitator check-ins before and during partner exercises; clear verbal and nonverbal stop signals; opt-out from any exercise without explanation; and facilitator decisions to pause, modify, or halt activities when distress or discomfort occurs. Touch intensity, touch location, and participation in specific partner exercises are adjusted based on participant comfort, boundaries, and emotional safety. The evaluation also incorporates feasibility-responsive tailoring through a flexible 4–6 week follow-up window and purposive recruitment of newer members (fewer than two prior classes) to support novelty and diversity in prior experience.

How well (planned):

Fidelity focus
This protocol is a program evaluation of an existing community-based intervention, so fidelity assessment is designed to capture: (a) whether core intervention features are present during delivery (consent-centered touch practices embedded in partner yoga) and (b) how much of the intervention participants receive (dose and attendance), while allowing real-world variability.

Strategies to support and monitor fidelity of core touch and consent elements
• Layered, ongoing consent is built into participation as a core intervention feature. Facilitators use brief verbal check-ins before and during partner-based exercises, and participants use clear verbal and nonverbal stop signals to pause or discontinue touch immediately.
• Facilitators receive training in trauma-informed communication, boundary-setting, and de-escalation strategies prior to implementation to support consistent delivery of consent-centered practices and appropriate responses to distress.
• Incidents of discomfort, withdrawal from activities, or boundary concerns are documented using structured field notes and reviewed during routine evaluation team meetings and monthly with the CAG and Studio leadership to support responsive adaptations and reinforce safety-related fidelity.

Dose and receipt measurement
• Attendance and retention will be measured using Studio attendance records (explicitly consented to by participants) to verify participation and quantify dose received (for example, number of classes attended during the observation period).
• Retention across baseline, post, and follow-up will be supported through reminders before each survey time point, timely delivery of incentives, flexible scheduling, affirming follow-up messages, and consistent updates about study progress.

Fidelity related to evaluation delivery (data collection quality)
• Instruments are co-developed with the CAG and the exploratory Comfort, Connection, and Touch Survey is pilot tested with CAG members and Studio facilitators for clarity and feasibility, with refinement planned before full implementation.

• Biweekly co-reflection sessions with Studio facilitators and leadership provide ongoing monitoring of implementation context and allow triangulation with participant-reported data.
• Data quality procedures include validation rules within survey platforms and periodic verification of exported datasets prior to analysis.
